# Solvability of the *p*-Adic Analogue of Navier–Stokes Equation via the Wavelet Theory

**DOI:** 10.3390/e21111129

**Published:** 2019-11-17

**Authors:** Ehsan Pourhadi, Andrei Khrennikov, Reza Saadati, Klaudia Oleschko, María de Jesús Correa Lopez

**Affiliations:** 1 International Center for Mathematical Modelling in Physics and Cognitive Sciences, Mathematical Institute, Linnaeus University, SE-351 95 Växjö, Sweden; ehsan.pourhadi.extern@lnu.se; 2 Department of Mathematics, Iran University of Science and Technology, Narmak, Tehran 16846-13114, Iran; rsaadati@eml.cc; 3 Centro de Geociencias, Campus UNAM Juriquilla, Universidad Nacional Autonoma de Mexico (UNAM), Blvd. Juriquilla 3001, 76230 Queretaro, Mexico; olechko@unam.mx; 4 Edificio Piramide, Boulevard Adolfo Ruiz Cortines 1202, Oropeza, 86030 Villahermosa, Tabasco, Mexico; maria.jesus.correa@pemex.com

**Keywords:** tree-like geometry, capillary networks, p-adic model of porous medium, fluid’s propagation, complex geological phenomena, p-adic analog of Navier–Stokes equation, pseudo-differential equations, p-adic wavelet basis, Schauder fixed point theorem, Vladimirov’s operator, existence of solution

## Abstract

*P*-adic numbers serve as the simplest ultrametric model for the tree-like structures arising in various physical and biological phenomena. Recently *p*-adic dynamical equations started to be applied to geophysics, to model propagation of fluids (oil, water, and oil-in-water and water-in-oil emulsion) in capillary networks in porous random media. In particular, a *p*-adic analog of the Navier–Stokes equation was derived starting with a system of differential equations respecting the hierarchic structure of a capillary tree. In this paper, using the Schauder fixed point theorem together with the wavelet functions, we extend the study of the solvability of a *p*-adic field analog of the Navier–Stokes equation derived from a system of hierarchic equations for fluid flow in a capillary network in porous medium. This equation describes propagation of fluid’s flow through Geo-conduits, consisting of the mixture of fractures (as well as fracture’s corridors) and capillary networks, detected by seismic as joint wave/mass conducts. Furthermore, applying the Adomian decomposition method we formulate the solution of the *p*-adic analog of the Navier–Stokes equation in term of series in general form. This solution may help researchers to come closer and find more facts, taking into consideration the scaling, hierarchies, and formal derivations, imprinted from the analogous aspects of the real world phenomena.

## 1. Introduction

The last decades have witnessed great use of Fourier and more generally wavelet analysis over the *p*-adic fields, and its various physical applications in physics, biology and cognitive science, and recently in geophysics. The keyword of these applications is “hierarchy”. These applications are based on representation of hierarchies by tree-like geometry. Hierarchy is also a natural attribute of ultrametric spaces which mathematically can be represented as a duality between ultrametric (non-Archimedean) spaces and trees of balls in these spaces where mathematical tools such as integral and series are frequently used (see also [1,2]). Thus, ultrametric (non-Archimedean) spaces play the crucial role in aforementioned applications. The simplest ultrametric spaces are given by homogeneous trees, *m*-adic trees, where m>1 is a natural number encoding the number of branches leaving each vertex of the tree. If m=p is a prime number, such ultrametric spaces can be endowed with the algebraic structure of a number field (addition, subtraction, multiplication, and division) that is denoted as $Q_{p},$ the field of *p*-adic numbers (Each number $x\in Q_{p}$ represents a branch of the *p*-adic tree. In the mathematical model, branches are infinite. Of course, trees in nature, e.g., capillary networks in random porous media, are finite. They are obtained as cutoffs of *p*-adic trees represented by $Q_{p},p>1$). This algebraic structure in combination with the ultrametric topology on $Q_{p}$ serve as the basis for analysis that have some similarity and a lot of dissimilarity with the real analysis (see, e.g., Escassut [3,4]).

The *p*-adic numbers were first applied in theoretical physics in an effort to solve one of the most remarkable problems of modern physics, that of combining quantum mechanics and gravitation theory. Hence it was conjectured in [5,6] that space-time geometry is non-Archimedean at Planck magnitudes ($∼10^{-33}$ cm). Regarding with diverse applications of the field $Q_{p}$ of *p*-adic numbers several later papers including applicable contents have been published, e.g., [7,8,9,10,11,12,13,14,15,16,17,18,19,20,21]. These applications in turn motivated the pure mathematicians to develop the new areas of *p*-adic analysis, containing *p*-adic wavelet theory (see, e.g., [22,23,24,25,26,27,28,29,30,31,32,33,34,35,36,37,38,39,40,41,42,43]).

In this paper, the solvability of the *p*-adic analog of the Navier–Stokes equation via the wavelet theory is discussed by the example of real world problem: the precise modeling of fluid flow in highly heterogeneous, multiscale, and anisotropic porous media with strongly hierarchical architecture. This problem is recognized among key technical challenges of Petroleum Industry looking for new analytical solutions of classical mathematical analogue and new type of computing perspectives, more closed to the pure science. The continuous interaction among rock, fluid, and flow properties, result in dynamics of the geometry of mass transfer and waves routes, especially their continuity and tortuosity, affected by strong mixture of a complex geological phenomena: tectonics, salt tectonics, carbonatation, fluid dynamics, including the turbulence. The joint physical, petrophysical, geological, and mathematical modeling in these conditions require the new typing of pores. In spite of classical pores division in families of fractures, vughs and micropores, we propose their joint typing as fluid/waves conduits, resulting from the complex mixture of different from the geological point of view, elemental flow units. The tree-like geometry of these multi-sized conduits can be described with high precision by *p*-adic numbers, which not only encode the scaling of real porous media space distribution, but also provide the modeling of fluid flow with industry-leading quality and resolution. This work is our new attempt to ensure the solvability of the *p*-adic analogue of Navier–Stakes equation via wavelet tools for real porous media. The further model, combining the *p*-adic analogue of conduits with multifractal modeling of flow and transport thorough these geometrically complex and strongly no lineal networks, just on the road. Under the general assumption of the thermodynamical nature of multifractal systems, we conclude that our trans-disciplinary approach is the example of the real world demands of the future of the Era of Big Data and Entropy, the Queen of the Unified Physical, Geological, Numerical and, in general, Mathematical analogical modeling.

**Figure 1:**
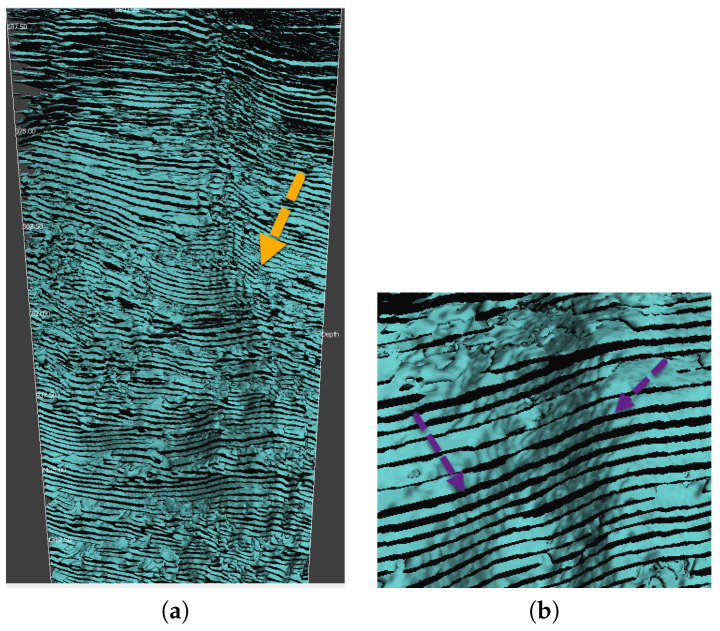
(**a**). The general view of Isosurface Cube constructed from the unfiltered seismic waves amplitudes, distributed across the oil reservoir. The tree-like signature of Geo-conduits is especially clear in the down part of the original image and is zoomed in on (**b**). The main question is to show that the same connected and tortuous Geo-conduits, which conducted the seismic waves, are also the guides of mass (oil) transfer in fractured reservoir of complex and unconventional architecture.

**Figure 2:**
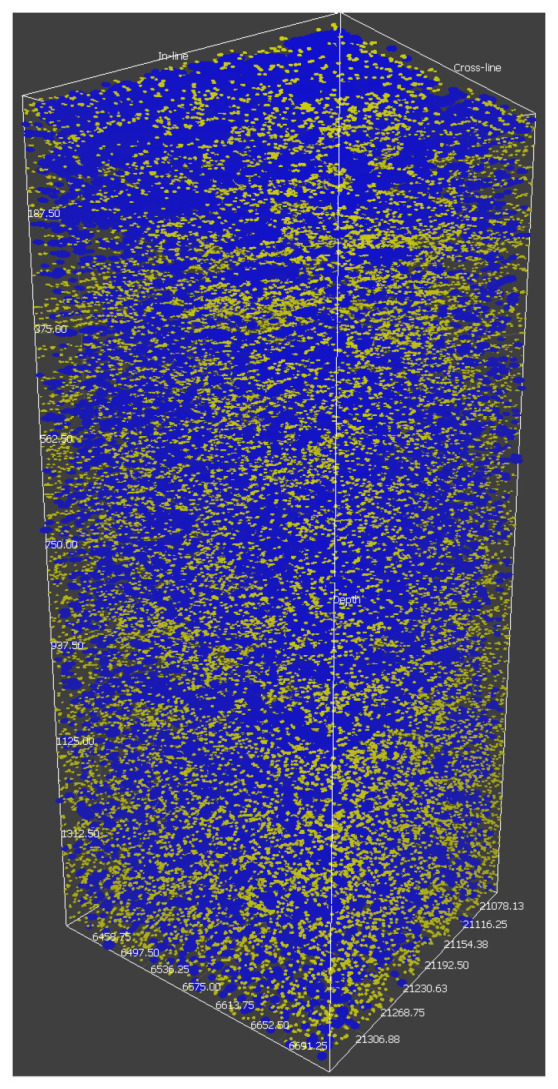
The effective metric for Geo-conduits patterns measurement was constructed by distribution of three sized balls within seismic cube.

Considering the historic remarks of this applied field, the cooperation between the research groups of K. Oleschko (applied geophysics and petroleum research) and A. Khrennikov (*p*-adic mathematical physics) led to initiating a new promising field of research [35,36,37,38]: *p*-adic and more generally ultrametric modeling of the dynamics of flows (of, e.g., water, oil, and oil-in-water and water-in-oil emulsion) in capillary networks in porous random media. The starting point of this project is the observation that tree-like capillary networks are very common geological structures, especially in carbonates (see Figure 1 and Figure 2). The latter serves as rock base of oil-reservoirs. Fluids propagate through such trees of capillaries, so it is useful to reduce the configuration space to these tree-like structures and the appropriate mathematical model of such a configuration space is defined by an ultrametric space.

In 2017, Oleschko et al. [38] focused the *p*-adic dynamics described by fractional differential operators (Vladimirov operators) starting with discrete dynamics based on hierarchically-structured interactions between the fluids’ volumes concentrated at different levels of the percolation tree and coming to the multiscale universal topology of the percolating nets. They presented a system of dynamical equations reflecting the tree structure of a capillary network in porous media and then derived the following nonlinear *p*-adic pseudo-differential equation for fluid’s velocity u(t.x) along capillaries:

(1) $$\begin{array}{c} \frac{\partial u(t,x)}{\partial t}=u\left(t,x\right)Du\left(t,x\right)-\theta D^{2}u\left(t,x\right)+G\left(t,x\right),\;x\in Q_{p},\;t,u\in R,t\geq 0, \end{array}$$

where θ is the viscosity parameter with the initial condition u(0,x)=φ(x). This equation can be considered as the *p*-adic analogue of the Navier–Stokes equation. We stress that this is just an analog of the Navier–Stokes equation. The tree-like configuration space differs crucially from the real space of hydrodynamics and this difference is reflected in dynamical equations. In particular, *u* is a real scalar and not a real vector with three coordinates as in the Navier–Stokes equation in the Euclidean space, see Section 3 for details. Alternative nonlinear term in *p*-adic analogue of the Navier–Stokes equation was considered in paper of Kozyrev [39]. Moreover, the mathematical theory of such equations has not yet been developed. Very recently, Khrennikov and Kochubei [43] investigated the local solvability of Equation (1) using the von Wahl’s theorem for the case G(t,x) as the source term vanishes.

In the present paper, inspired by [43], our attention will be turned exclusively to study the solvability of Equation (1) using the technique of wavelet basis and a well-known fixed point theorem. Theory of *p*-adic wavelets was initiated by Kozyrev [11] and it found numerous applications (see, e.g., [2,12,13,35]), including modeling of fluid propagation in capillary networks in random disordered medium [36,37,38]. In Section 4, under certain conditions we study the solvability of an infinite system which is derived from (1) (with wavelet expansion of solution) in the sequence space $c_{0}$. In Section 5, we employ a numerical method, the so-called Adomian decomposition method (ADM), to formulate the solution of Equation (1) represented by series.

This paper demonstrates that, for fluids’ propagation through capillary networks in porous disordered media, *p*-adic linear models developed and investigated in our previous works [35,36] can be successfully generalized (at the mathematical level of rigorousness) to nonlinear phenomena.

## 2. Geophysics: From Fractal to Tree-Like Models

The starting point of our research was fractal/multifractal modeling in geophysics [44,45]. The detailed presentation on such an approach can be found in Section 1.1 of our paper [36]. Here we briefly point to the most important moments. The fractal/multifractal scaling features of capillary networks was studied (both theoretically and experimentally) since the early 1980s and 1990s [46,47]: for invasion percolation, diffusion-limited aggregation (DLA), anti-DLA processes [48].

Later fractal modeling of fluids’ flows in porous random media [49], including transport through tree-like networks and diffusion on fractals [50] was widely used in oil recovery studies [51]. This modeling was supported by theoretical and experimental studies demonstrating that fluid’s flow through tree-like networks is faster [52,53].

Stanley and Meakin [46] have discussed the important thermodynamics aspects of multifractality in physics and chemistry, founding the formal analogy among the probability distribution function Z(q) and partition function Z(β). Therefore, the analogy between the Legendre transform f(α) and entropy *H*, as well as between the function α and energy *E* was found. These analogies are the key points for physics of fractal capillaries patterns treelike morphology. The difference in the medium heterogeneity can be quantified by several multifractal indicators (for instance, the degree of the graph symmetry or strength of singularity).

Thus, fractal/multifractal studies led to an understanding of the importance of tree-like structures in mathematical modeling of fluids’ flows through capillary networks in porous disordered media.

## 3. Navier—Stokes Equation on Tree-Like Configuration Space and Its Generalizations

Here we briefly repeat the basics of the physical model leading to the *p*-adic analog of the Navier–Stokes equation, see [38] for details. The main motivation for derivation of this equation is application to modeling propagation of fluid’s flow through Geo-conduits, consisting of the mixture of fractures (as well as fracture’s corridors) and capillary networks, detected by seismic as joint wave/mass conducts (see Figure 1 and Figure 2).

In such modeling [35,36], the tree-like structure of capillaries in real porous disordered media is represented by trees endowed with the root distance—ultrametric spaces. The rock environment of capillary networks is ignored. We explore only geometry of the network of capillaries. So, instead of the Euclidean configuration space, we use the tree-like configuration space. The rock environment is encoded in the coefficients of the dynamical equations describing fluids’ propagation through capillary networks in porous media. Tree-like geometry and ultrametric spaces give the proper mathematical model for such networks.

In our model, the “spatial-variable” *x* belongs to an ultrametric space denoted by symbol *X* and the time variable *t* is real. We operate with functions f(t,x) depending on real and ultrametric variables. Here *x* is the “pore network coordinate”, each pathway of pore capillaries is encoded by a point *x* of the ultrametric space (or in the tree-like representation—by a branch of the tree, see [36] for details). Time is usual real time. Thus, by assigning the ultrametric coordinate *x* to a system (e.g., oil or water droplet) we know in which pathway composed of capillaries this system is located. The ultrametric model provides a fuzzy description of system’s location in a pore network. The simplest trees are homogeneous trees, the *p*-adic trees. Furthermore, in this paper, we concentrate our study on such configuration spaces. The general ultrametric case was considered in [36]. Of course, real capillary structures are described by non-homogeneous trees. However, the general case is essentially more complex mathematically. Here we were able to study (at the mathematical level of rigorousness) only linear pseudo-differential equations for fluid’s propagation through capillary networks in porous media.

Now, we turn to derivation of the *p*-adic analog of the Navier–Stokes equation, for simplicity we consider the case p=2. Consider the homogeneous tree with two branches leaving each vertex. The root of the tree is denoted by $I_{0};$ the levels of the tree are enumerated, n=0,1,2,‥‥, where n=0 corresponds to the root $I_{0}.$ Vertexes at the *n*th level of the tree are enumerated as $I_{n,j},j=1,\ldots ,2^{n}.$ We are interested in capillaries connecting successive vertexes, i.e., from $I_{n-1,j}$ to the corresponding two vertexes at level n. They can be labeled in the same way as corresponding vertices, i.e., $E_{1,1}=I_{0}I_{1,1}$ and $E_{1,2}=I_{0}I_{1,2},$ the capillaries going from root $I_{0}$ to vertexes $I_{1}$ and $I_{2}.$ There are 2*n* edges connecting vertexes of the (n−1)-th level with vertices of the *n*-th level:

$$E_{n,1}=I_{n-1,1}I_{n,1},\;E_{n,2}=I_{n-1,1}I_{n,2},\;E_{n,3}=I_{n-1,2}I_{n,3},\;E_{n,4}=I_{n-1,2}I_{n,4},‥,$$

or

$$E_{n,2j-1}=In-1,jI_{n,2j-1},\;E_{n,2j}=I_{n-1,j}I_{n,2j}.$$

In the present model the diameters and lengths of edges are coupled by scaling 1/2. Thus at each vertex $I_{n,j}$ fluid’s flow is split into two capillaries that are two times shorter and thinner than the capillary incoming to this vertex. Denote by $u_{n,j}\left(t\right)$ the average velocity of fluid along capillary $E_{n,j}.$ Thus, in our model we are not interested in fluid’s velocity in each point of a capillary, but only in its average with respect to capillary’s volume. The velocity is a real number. Its sign encodes the (average) direction of the velocity along the capillary, towards and backwards with respect to the root of the tree.

In the mathematical model, instead of the discrete variables n,j we can operate with the continuous 2-adic variable $x\in Q_{2}$ and velocity u(t,x), where $t,u\in R,x\in Q_{2}.$ In [38], we derived nonlinear pseudo-differential dynamical Equation (1) for velocity u(t,x). Its form is analogous to the form of the standard Navier–Stokes equation. However, it is the scalar-function equation, since fluid’s propagation is only along capillary’s axis.

Derivation of the *p*-adic analog of the Navier–Stokes equation opens the door to consideration of a bunch of interesting problems on dynamics of fluids in capillary networks. One of such problems is coupling of the dynamics of fluid with electromagnetic field, derivation of the *p*-adic (capillary network) analog of the system of magneto-fluid dynamics equations (cf. [54]). The main difficulty is derivation of “restriction” of the Maxwell equations onto the capillary network and then their coupling with the *p*-adic Navier–Stokes equation (cf. [54]). Such a theory can find applications in geophysics (cf. [55]).

Finally, we remark that the tree-like structure of capillary networks is not only very common in nature, but recently they started to be artificially manufactured—to speed up fluid’s propagation (see [56] and references herein). This applied research is theoretically justified by the works of Shou et al. [52,53] on the tree-like basis of acceleration of fluid’s flows (see also [57] on *p*-adic modeling). Such industrial applications stimulate *p*-adic (and more general ultrametric) modeling of fluids’ propagation in tree-like capillary networks.

## 4. Mathematical Preliminaries

In this section, we recall some auxiliary facts concerned with *p*-adic fields and wavelet theory. In view of the Ostrovski theorem (see [6], Ch. I, § 1.1), there exists, in some sense, only two “universes” of equal status: the real universe and the *p*-adic one. The real “universe” is structured by the field of real numbers R, which is introduced by the completion of the field Q of rational numbers with relevance to the usual Euclidean norm, and the *p*-adic “universe” is based on the field $Q_{p}$ of *p*-adic numbers, which is given as the completion of the field Q with respect to the *p*-adic norm ${\left|\cdot \right|}_{p}$. This norm is defined as below. ${\left|\cdot \right|}_{p}:{\left|0\right|}_{p}=0$; if an arbitrary rational number x≠0 is represented as $x=p^{\gamma }\frac{m}{n}$ uniquely, where γ=γ(x)∈Z and m,n are not divisible by *p* then ${\left|x\right|}_{p}=p^{-\gamma }$. This norm satisfies the following properties:

(i) ${\left|x\right|}_{p}\geq 0$ for every $x\in Q_{p}$, and ${\left|x\right|}_{p}=0$ if and only if x=0;
(ii) ${\left|xy\right|}_{p}={\left|x\right|}_{p}{\left|y\right|}_{p}$ for every $x,y\in Q_{p}$;
(iii) ${\left|x+y\right|}_{p}\leq {\max\{|x|}_{p}{,|y|}_{p}\},$ for every $x,y\in Q_{p}$, and when ${\left|x\right|}_{p}\neq {\left|y\right|}_{p}$, we have ${\left|x+y\right|}_{p}={\max\{|x|}_{p}{,|y|}_{p}\}$.

The condition (iii) as the strong triangle inequality makes the norm ${\left|\cdot \right|}_{p}$ non-Archimedean and hence the space $\left(Q_{p},|\cdot {|}_{p}\right)$ is an ultrametric space.

We shall systematically utilize the notation and results from [6]. Denote by N,Z,C the sets of positive integers, integers, and complex numbers, respectively.

Any *p*-adic number $x\in Q_{p}$, x≠0, is represented in the canonical form as follows

(2) $$\begin{array}{c} x=\sum_{j=\gamma }^{\infty }x_{j}p^{j} \end{array}$$

where γ=γ(x)∈Z, and $x_{k}=0,1,\ldots ,p-1,\;x_{0}\neq 0,\;k=0,1,\ldots$ The series converges in the *p*-adic norm ${\left|\cdot \right|}_{p}$ to $p^{-\gamma }$, that is, ${\left|x\right|}_{p}=p^{-\gamma }$. Hence, the absolute value ${\left|\cdot \right|}_{p}$ takes the discrete set of nonzero values $p^{\gamma }$, for γ∈Z. The fractional part of a *p*-adic number $x\in Q_{p}$ given by (2) is defined as

(3) $$\begin{array}{c} {\left\{x\right\}}_{p}=\left\{\begin{array}{c} 0,\;\mathrm{if}\;\gamma (x)\geq 0\;\mathrm{or}\;x=0,\\ p^{\gamma }\left(x_{0}+x_{1}p+x_{2}p^{2}+\cdots +x_{|\gamma |-1}p^{|\gamma |-1}\right),\;\mathrm{if}\;\gamma \left(x\right)<0. \end{array}\right. \end{array}$$

The additive character $\chi_{p}$ of the field $Q_{p}$ is given by

$$\chi_{p}\left(x\right)=e^{2\pi i{\left\{x\right\}}_{p}},\;x\in Q_{p}.$$

The topology equipped with ${\left|\cdot \right|}_{p}$ in $Q_{p}$ are known by

$$B_{\gamma }\left(a\right)=\{x\in Q_{p}{:|x-a|}_{p}\leq p^{\gamma }\},\;S_{\gamma }\left(a\right)=\{x\in Q_{p}{:|x-a|}_{p}=p^{\gamma }\}$$

as balls and spheres of radius $p^{\gamma }$ with center at *a*, respectively. It is worth mentioning that any point of the ball is its center, besides, any two balls in $Q_{p}$ are either disjoint or one is included in the other. Furthermore, all balls and spheres are simultaneously open and closed sets in $Q_{p}$. For the certain case, the unit ball $Z_{p}=B_{0}\left(0\right)$ is the ring of *p*-adic integers consisting of the elements represented by the sum of *p* mutually disjoint balls.

The topological group $\left(Q_{p},+\right)$ is locally compact commutative and thus there is a additive Haar measure dx, which is positive and invariant under the translation, i.e., $d\left(x+a\right)=dx,a\in Q_{p}$. This measure is unique by normalizing dx so that

$$\int_{B_{0}}dx=1,\;d\left(ax+b\right)={\left|a\right|}_{p}dx,\;a\in Q_{p}^{*}=Q_{p}-\left\{0\right\}.$$

Regarding with the additive normalized character $\chi_{p}\left(x\right)$ on $Q_{p}$ we get

$$\int_{B_{\gamma }}\chi_{p}\left(\xi x\right)dx=p^{\gamma }\Omega (p^{\gamma }{\left|\xi \right|}_{p}),$$

where Ω(t) is the characteristic function of the segment [0,1]⊂R.

A complex-valued function *f* in $Q_{p}$ is said to be a *locally constant function* if for any $x\in Q_{p}$, there exists an integer l(x)∈Z such that f(x+y)=f(x), for every $y\in B_{l(x)}$. The largest of these numbers, l=l(f), is called the *parameter of constancy* of the function f. We denote the space of locally constant functions on $Q_{p}$ by $E(Q_{p})$. Indicate by $D(Q_{p})$ the space of Bruhat–Schwartz test functions, i.e., the subspace of $E(Q_{p})$ including compactly supported functions. Moreover, denote by $D^{\prime }\left(Q_{p}\right)$ the set of all linear functionals on $D(Q_{p})$ (see also ([6], VI.3)).

The Fourier transform of test function $\varphi \in D(Q_{p})$ is given by the formula

$$\hat{\varphi }\left(\xi \right)=F\left[\varphi \right]\left(\xi \right)=\int_{Q_{p}}\varphi \left(x\right)\chi_{p}\left(\xi x\right)dx,\;\xi \in Q_{p}.$$

This means $\hat{\varphi }\left(\xi \right)\in D\left(Q_{p}\right)$ and $\varphi \left(x\right)=F^{-1}\left[\varphi \right]\left(x\right)=\int_{Q_{p}}\hat{\varphi }\left(\xi \right)\chi_{p}\left(-\xi x\right)d\xi$ as the inverse Fourier transform.

Consider $L^{2}\left(Q_{p}\right)$ as the set of measurable C-valued functions *f* on $Q_{p}$ such that

$${∥f∥}_{L^{2}\left(Q_{p}\right)}={\left(\int_{Q_{p}}{\left|f\left(x\right)\right|}^{2}dx\right)}^{\frac{1}{2}}<\infty$$

which is evidently a Hilbert space with the inner product

$$\langle f,g\rangle =\int_{Q_{p}}f\left(x\right)\bar{g(x)}dx,\;f,g\in L^{2}\left(Q_{p}\right),$$

and ${∥f∥}_{L^{2}\left(Q_{p}\right)}^{2}=\langle f,f\rangle .$

This guarantees a linear isomorphism taking $D(Q_{p})$ onto $D(Q_{p})$. It can be uniquely extended to a linear isomorphism of $L^{2}\left(Q_{p}\right)$. Moreover, the Plancherel equality holds

$$\langle f,g\rangle =\langle \hat{f},\hat{g}\rangle ,\;f,g\in L^{2}\left(Q_{p}\right).$$

### 4.1. p-Adic Wavelet Theory

Throughout this section, we gather some facts related with the theory of *p*-adic wavelets which is widely employed in so many applications. It is now hard to find an area of engineering where wavelets are not applied. In 1910, Haar [58] initially presented the wavelet basis by an orthonormal basis in $L^{2}\left(Q_{p}\right)$ including dyadic translations and dilations of a single function; since then various generalizations of it have been revealed in several results. It is interesting to know that it took almost a century to create another wavelet function whose shifts and dilations would bring an orthogonal basis. The intensive progression in wavelet theory initiated only in the 1990s. At that moment Meyer [59] and Mallat [60,61] improved a scheme of structure for wavelet functions based on the concept of multiresolution analysis (MRA); see, for instance, [62], Ch. 5, [63], § 2.1.

Regarding with the theory of *p*-adic wavelets, it has a short background in comparison with that in the real status. In 2002, Kozyrev [11] found a compactly supported *p*-adic wavelet basis, similar to the real Haar basis, for $L^{2}\left(Q_{p}\right)$. Kozyrev’s wavelet functions have the following structure:

(4) $$\begin{array}{c} \psi_{k}\left(x\right)=\chi_{p}\left(p^{-1}kx\right){\Omega (|x|}_{p}),\;x\in Q_{p}. \end{array}$$

where $\chi_{p}$ and Ω are the standard additive character of $Q_{p}$ and characteristic function of [0,1], respectively.

This wavelet basis (created by the shifts and dilations of the wavelet functions (4)) contains of the wavelet functions

(5) $$\begin{array}{c} \psi_{k;jn}\left(x\right)=p^{-\frac{j}{2}}\chi_{p}\left(p^{-1}k\cdot \left(p^{j}x-n\right)\right)\Omega (|p^{j}x-n{|}_{p}),\;x\in Q_{p} \end{array}$$

where $k\in J_{p}=\left\{1,2,\ldots ,p-1\right\}$, j∈Z, and *n* is taken as an element of the *m*-direct product of factor group

$$Q_{p}/Z_{p}=\left\{\sum_{i=a}^{-1}n_{i}p^{i}\;|\;n_{i}=0,1,\ldots ,p-1,\;a\in Z^{-}\right\}.$$

That is, *n* belongs to $\{x\in Q_{p}:\;{\left\{x\right\}}_{p}=x\}.$

### 4.2. Vladimirov’s Operator and p-Adic Lizorkin Spaces

Introduced by V.S. Vladimirov [6], pseudo-differential operator *A* (on the field of *p*-adic numbers) in an open set $O\subset Q_{p}$ is given by

(6) $$\begin{array}{c} \left(A\varphi \right)\left(x\right)=\int_{Q_{p}}A\left(\xi ,x\right)\hat{\varphi }\left(\xi \right)\chi_{p}\left(-\xi x\right)d\xi ,\;x\in O \end{array}$$

which acts on C-valued functions φ(x) of *p*-adic arguments x∈O. Here we assume that functions φ(x) are extended by zero from the set O on whole space $Q_{p}$, and $\hat{\varphi }\left(\xi \right)$ are their Fourier transforms recalled previously. The function $A\left(\xi ,x\right),\xi \in Q_{p},x\in O$ is called *symbol* of the operator A.

In [64,65] Lizorkin presented spaces invariant under the real actions of fractional operators. These spaces can be defined in *p*-adic case. In view of [25,66], the *p-adic Lizorkin space* of test functions is described as follows:

$$\Phi =\Phi \left(Q_{p}\right)=\left\{\phi :\;\phi =F\left[\psi \right],\;\psi \in \Psi \right\}.$$

such that

$$\Psi =\Psi \left(Q_{p}\right)=\left\{\psi \in D\left(Q_{p}\right),\;\psi \left(0\right)=0\right\}.$$

Clearly, Ψ,Φ≠∅. Regarding the fact that Fourier transform is a linear isomorphism $D(Q_{p})$ into $D(Q_{p})$, one can see that $\Psi ,\Phi \in D(Q_{p})$. The space $\Phi (Q_{p})$ can be decorated with the topology of the space $D(Q_{p})$, which turns it into a complete space. The space Φ can be determined by the following characterization:

ϕ∈Φ if and only if $\phi \in D(Q_{p})$ and

$$\int_{Q_{p}}\phi \left(x\right)dx=0.$$

In addition, the space $\Phi^{\prime }=\Phi^{\prime }\left(Q_{p}\right)$ as the topological dual of Φ is said to be the Lizorkin space of *p*-adic distributions (see also [25]).

The Vladimirov operator $D^{\alpha }$ (initially introduced by Taibleson) also is included in the class (6) with symbol $A\left(\xi \right)={\left|\xi \right|}_{p}^{\alpha }$, i.e.,

(7) $$\begin{array}{c} \left(D^{\alpha }f\right)\left(x\right)=F^{-1}{\left[|\cdot \right|}_{p}^{\alpha }F\left[f\right]\left(\cdot \right)]\left(x\right),\;f\in \Phi^{\prime }\left(Q_{p}\right), \end{array}$$

where α∈C. The formula (7) can be rewritten as a convolution of the following functions:

$$\left(D^{\alpha }\varphi \right)\left(x\right)=\kappa_{-\alpha }\left(x\right)*\varphi \left(x\right)=\langle \kappa_{-\alpha }\left(x\right),\varphi \left(x-\xi \right)\rangle ,\;\varphi \in \Phi^{\prime }\left(Q_{p}\right),\;\alpha \in C$$

where the distribution $\kappa_{\alpha }\in \Phi^{\prime }\left(Q_{p}\right)$ is called the *Riesz kernel* given by

$$\begin{array}{c} \kappa_{\alpha }\left(x\right)=\left\{\begin{array}{c} \frac{{\left|x\right|}_{p}^{\alpha -1}}{\Gamma_{p}\left(\alpha \right)},\;\mathrm{if}\;\alpha \neq 0,1,\\ \delta (x),\;\mathrm{if}\;\alpha =0,\\ \frac{p^{-1}-1}{\log p}\log{\left|x\right|}_{p},\;\mathrm{if}\;\alpha =1, \end{array}\;x\in Q_{p}\right. \end{array}$$

and $\Gamma_{p}\left(\alpha \right)=\frac{1-p^{\alpha -1}}{1-p^{-\alpha }}$ is the Γ-function (for more details see [6]).

The domain of $D^{\alpha }$ is given by

$$M\left(D^{\alpha }\right)=\left\{\varphi \in L^{2}\left(Q_{p}\right)\;|\;D^{\alpha }\varphi \in L^{2}\left(Q_{p}\right)\right\}.$$

We remark that all the concepts as above can be reconsidered in multidimensional *p*-adic field $Q_{p}^{n}$ which is not in our considerations in the current paper.

## 5. Solvability of the p-Adic Navier–Stokes Equation

This section deals with the main result of our paper. Namely, we will apply the wavelet and fixed point theories to show the existence of solutions for our problem. Indeed, we study the existence of solution of the *p*-adic pseudo-differential equation in [0,∞) given as (1).

Let us assume there exists

$$u\in U_{I}:=C^{1}\left(I,R\right)\cap C\left(I,M\left(D^{1}\right)\right)\cap C\left(I,M\left(D^{2}\right)\right),$$

for any interval *I* in terms of wavelet functions $\psi_{k;jn}\left(x\right)$ with coefficients $u_{k;jn}\left(t\right)$, that is,

(8) $$\begin{array}{c} u\left(x,t\right)=\sum u_{k;jn}\left(t\right)\psi_{k;jn}\left(x\right),\;\varphi_{k;jn}:=u_{k;jn}\left(0\right)=\langle \varphi \left(x\right),\psi_{k;jn}\left(x\right)\rangle . \end{array}$$

Moreover,

(9) $$\begin{array}{c} \begin{array}{c} \frac{\partial u(x,t)}{\partial t}=\sum u_{k;jn}^{\prime }\left(t\right)\psi_{k;jn}\left(x\right),\;D_{x}^{\alpha }u\left(x,t\right)=\sum p^{\alpha (1-j)}u_{k;jn}\left(t\right)\psi_{k;jn}\left(x\right). \end{array} \end{array}$$

On the other hand, taking into account that

(10) $$\begin{array}{c} \left[uDu\right]\left(x,t\right)=\sum_{ı\in J}F_{ı}\left(t,\hat{u}\right)\psi_{ı}\left(x\right),\;\hat{u}={\left(u_{ı}\right)}_{ı},\;ı=\left(k,j,n\right)\in J:=J_{p}\times Z\times Q_{p}/Z_{p}, \end{array}$$

it implies that

(11) $$\begin{array}{c} F_{ı}\left(t,\hat{u}\right)=\langle \left[uDu\right]\left(x,t\right),\psi_{ı}\left(x\right)\rangle =\langle \sum_{(k,j,n)\in J}p^{1-j}|u_{k;jn}\left(t\right){|}^{2}\Omega (|p^{j}{x-n|}_{p}),\psi_{ı}\left(x\right)\rangle :=F_{ı}\left(\hat{u}\right). \end{array}$$

Replacing (8), (9) and (10) in Equation (1) and regarding the action of Vladimirov operator on *u* we derive the following infinite system:

(12) $$\begin{array}{c} u_{ı}^{\prime }\left(t\right)=F_{ı}\left(\hat{u}\right)-\theta \cdot p^{2(1-j)}u_{ı}+G_{ı}\left(t\right):=F_{ı}\left(\hat{u}\right)+G_{ı}\left(t\right), \end{array}$$

where $G_{ı}\left(t\right)=\langle G\left(t,x\right),\psi_{ı}\left(x\right)\rangle$. Removing the index *ı* and fixing *j* yields the following equation

(13) $$\begin{array}{c} {\hat{u}}^{\prime }\left(t\right)=F\left(\hat{u}\right)-\theta \cdot p^{2(1-j)}\hat{u}+G\left(t\right):=F\left(\hat{u}\right)+G\left(t\right). \end{array}$$

Generally, the solution of this nonlinear differential equation cannot be presented explicitly but for the special cases we have:

- If G(t,x)≡0, then
  $$\begin{array}{c} H_{ı}\left(\hat{u}\right):=\int \frac{du_{ı}}{F_{ı}\left(\hat{u}\right)}=t+c, \end{array}$$
  and using the initial condition we see that $c=H_{ı}\left({\left(\varphi_{ı}\right)}_{ı}\right)$, that is, the solution of Equation (1) takes the following form
  $$\begin{array}{c} u\left(x,t\right)=\sum_{ı\in J}u_{ı}\left(t\right)\psi_{ı}\left(x\right)\;\mathrm{where}\;H_{ı}\left(\hat{u}\left(t\right)\right)=t+H_{ı}\left(\hat{\varphi }\right),\;\hat{u}={\left(u_{ı}\right)}_{ı},\;\hat{\varphi }={\left(\varphi_{ı}\right)}_{ı}. \end{array}$$
  Furthermore, if $H_{ı}$ is invertible, then
  $$\begin{array}{c} u\left(x,t\right)=\sum_{ı\in J}{\left(H_{ı}^{-1}\left(t+H_{ı}\left(\hat{\varphi }\right)\right)\right)}_{ı}\psi_{ı}\left(x\right). \end{array}$$
- If $F\left(\hat{u}\right)=A\hat{u}$ and G(t)=Bt then the solution is represented in the parametric form:
  $$\begin{array}{c} t=\int {\left(A\tau +B\right)}^{-1}d\tau +C,\;\hat{u}={\left(\frac{1}{A}\left[\tau -B\int {\left(A\tau +B\right)}^{-1}d\tau +BC\right]\right)}_{ı}. \end{array}$$

To find more cases of Equation (13) we refer the reader to see ([67], [Section 1.6.3]).

In the sequel, we focus on the solvability of Equation (13) in the general form.

## 6. Solvability of Infinite System (12) over the Sequence Space $c_{0}$

Let us first convert the infinite system of differential equations (12) into the following infinite system of integral equations

(14) $$\begin{array}{c} u_{ı}\left(t\right)-\varphi_{ı}=\int_{0}^{t}F_{ı}\left(s,u_{{ı}_{1}}\left(s\right),u_{{ı}_{2}}\left(s\right),\ldots \right)ds+G_{ı}\left(t\right),\;\mathrm{where}\;G_{ı}\left(t\right)=\int_{0}^{t}G_{ı}\left(t\right)dt,\;ı\in J. \end{array}$$

The existence theory concerning the infinite systems of integral equations is satisfactorily developed up to now and we are interested in study the system (14) in the Banach sequence space $c_{0}$ containing sequences of real numbers converging to zero. We recall that $c_{0}$ is a closed subspace of $c\subset \ell_{\infty }$ as the space of convergent sequences. It turns out this space is very convenient and natural for investigations of infinite systems both differential and integral equations. In what follows, we proceed our study in the Banach space $c_{0}$ including of real sequences converging to zero with the standard norm $∥\hat{u}{∥}_{c_{0}}=\left\{\right|u_{ı}\left|:\;ı\in J\right\}$ for $\hat{u}={\left(u_{ı}\right)}_{ı}$. Note that the index has been altered following our notation and the subjected problem.

In the following we intend to apply the generalized theorem of Arzéla (see also [68]) which describes a criterion of compactness in the space C(I,E) for the arbitrary interval *I* and the Banach space *E*.

**Theorem** **1.** *A bounded subset U of the space C(I,E) is relatively compact if and only if all functions belonging to U are equicontinuous on I and the set U(t):={u(t):u∈U} is relatively compact in E for each t∈I.*

It is worth mentioning that a bounded subset *U* of $c_{0}$ is relatively compact if and only if

$$\lim_{|ı|\to \infty }\left[\underset{u\in U}{\sup}\left[\max\{\right|u_{ȷ}\left|:\;ı\leq ȷ\}\right]\right]=0.$$

Suppose that $G_{I}:=\max_{t\in I}\left|G\left(t\right)\right|<\infty$ and the interval I=[0,T] for T>0 is given. To investigate the solvability of nonlinear pseudo-differential Equation (1) it only needs to focus on the existence of $\hat{u}\left(t\right)$ from the system (14). To do this, let us first present the following well-known fixed point result.

In what follows, system (14) will be investigated under the following hypotheses.

(i) The functions $F_{ı}$ are given on the set $I\times R^{\infty }$ and take real values (ı∈J). Further, the operator $\tilde{F}$ is defined on the space $I\times c_{0}$ in the following way:
  $$\left(t,\hat{u}\right)⟼\left(\tilde{F}\hat{u}\right)\left(t\right)=\left(F_{{ı}_{1}}\left(t,\hat{u}\right),F_{{ı}_{2}}\left(t,\hat{u}\right),\ldots \right)$$
  which maps the space $I\times c_{0}$ into $c_{0}$ and is such that the class of all functions ${\left\{\left(\tilde{F}\hat{u}\right)\left(t\right)\right\}}_{t\in I}$ is equicontinuous at every point of the space $c_{0}$.
(ii) There exist nonnegative functions $\alpha_{ı}\left(t\right)$ and $\beta_{ı}\left(t\right)$ defined, integrable and uniformly bounded on *I* and such that $\int_{0}^{T}\beta_{ı}\left(s\right)ds<1$. Furthermore, the function sequence $\left(\int_{0}^{t}\alpha_{ı}\left(s\right)ds\right)$ converges monotonically to zero at each point t∈I while the function sequence $\left(\int_{0}^{t}\beta_{ı}\left(s\right)ds\right)$ is non-increasing at each point t∈I and the following estimate is satisfied:
  $$|F_{ı}\left(t,u_{{ı}_{1}},u_{{ı}_{2}},\ldots \right)|\leq \alpha_{ı}\left(t\right)+\beta_{ı}\left(t\right)\cdot \sup\{|u_{ȷ}\left|:\;ı\leq ȷ\right\}$$
  for each t∈I, ı∈J and for each $\hat{u}={\left(u_{ı}\right)}_{ı}\in c_{0}$.
(iii) The functions $G_{ı}+\varphi_{ı}:I\to R$ are continuous on *I* and the sequence $\left(\right|G_{ı}\left(t\right)+\varphi_{ı}{\left|\right)}_{ı}$ converges monotonically to zero at each point t∈I.

In the following we recall the well-known Schauder fixed point theorem which is crucial to present our result.

**Theorem** **2** (Schauder Fixed Point Theorem ([69], [Theorem 4.1.1]))**.** *Let U be a nonempty and convex subset of a normed space E. Let T be a continuous mapping of U into a compact set K⊂U. Then T has a fixed point.*

Now we can formulate our main result.

**Theorem** **3.** *Under the assumptions (i)-(iii), the infinite system *(14)* has at least one solution $\hat{u}\left(t\right)={\left(u_{ı}\left(t\right)\right)}_{ı}$ such that $\hat{u}\left(t\right)\in c_{0}$ for each t∈I.*

**Proof.**  Indicate by S the subset of space $C(I,c_{0})$ including all functions $\hat{u}\left(t\right)={\left(u_{ı}\left(t\right)\right)}_{ı}$ so that

$$\begin{array}{c} \sup\{|u_{ȷ}\left(t\right)\left|:\;ı\leq ȷ\right\}\leq a_{ı}\left(t\right)+b_{ı}\left(t\right) \end{array}$$

for ı∈J and t∈I, where ≤ in index is the usual partial order in J, and $a_{ı}\left(t\right)$, $b_{ı}\left(t\right)$ are defined in the following way:

$$\begin{array}{c} a_{ı}\left(t\right)=\frac{\int_{0}^{t}\alpha_{ı}\left(s\right)ds}{\left(1-\int_{0}^{t}\beta_{ı}\left(s\right)ds\right)},\;b_{ı}\left(t\right)=\frac{\sup\{|G_{ı}\left(s\right)+\varphi_{ı}|:\;0\leq s\leq t\}}{\left(1-\int_{0}^{t}\beta_{ı}\left(s\right)ds\right)}, \end{array}$$

for ı∈J.

Remark that the functions $a_{ı}\left(t\right)$ and $b_{ı}\left(t\right)$ are nondecreasing functions on the interval *I* and non-increasing sequences. Besides, from the assumptions, it follows that the functional sequences $\left(a_{ı}\left(t\right)\right)$ and $\left(b_{ı}\left(t\right)\right)$ converge uniformly on *I* to the function vanishing identically on *I*.

Let us assume the operator Γ defined on the space $C(I,c_{0})$ as follows:

$$\begin{array}{c} \left(\Gamma \hat{u}\right)\left(t\right)=\left({\left(\Gamma \hat{u}\right)}_{ı}\left(t\right)\right)=\left(\varphi_{ı}+\int_{0}^{t}F_{ı}\left(s,u_{{ı}_{1}}\left(s\right),u_{{ı}_{2}}\left(s\right),\ldots \right)ds+G_{ı}\left(t\right)\right). \end{array}$$

Notice that the operator Γ maps the set S into itself. Indeed, fix arbitrarily *ı* and $\hat{u}\left(t\right)\in S$. Then for ȷ≥ı, we get

$$\begin{array}{c} \begin{array}{cc} |{\left(\Gamma \hat{u}\right)}_{ȷ}\left(t\right)| & \leq |G_{ȷ}\left(t\right)+\varphi_{ȷ}\left|+\right|\int_{0}^{t}F_{ȷ}\left(s,u_{{ı}_{1}}\left(s\right),u_{{ı}_{2}}\left(s\right),\ldots \right)ds|\\ & \leq |G_{ı}\left(t\right)+\varphi_{ı}|+\int_{0}^{t}[\alpha_{ȷ}\left(s\right)+\beta_{ȷ}\left(s\right)\cdot \sup\{|u_{ı}\left(s\right)|:\;ȷ\leq ı\}]ds\\ & \leq |G_{ı}\left(t\right)+\varphi_{ı}|+\int_{0}^{t}\alpha_{ȷ}\left(s\right)ds+\int_{0}^{t}\beta_{ȷ}\left(s\right)\left[a_{ȷ}\left(s\right)+b_{ȷ}\left(s\right)\right]ds\\ & \leq |G_{ı}\left(t\right)+\varphi_{ı}|+\int_{0}^{t}\alpha_{ı}\left(s\right)ds+\int_{0}^{t}\beta_{ı}\left(s\right)\left[a_{ı}\left(s\right)+b_{ı}\left(s\right)\right]ds\\ & \leq \sup\{|G_{ı}\left(s\right)+\varphi_{ı}\left|:\;0\leq s\leq t\right\}+\int_{0}^{t}\alpha_{ı}\left(s\right)ds+\left(a_{ı}\left(t\right)+b_{ı}\left(t\right)\right)\int_{0}^{t}\beta_{ı}\left(s\right)ds\\ & \leq a_{ı}\left(t\right)+b_{ı}\left(t\right). \end{array} \end{array}$$

Now we prove that the operator Γ is continuous on the set S.

Consider ϵ>0 arbitrarily fixed and ${\hat{u}}_{0}\in S$. Then, taking into account the equicontinuity of the family of functions revealed in assumption (i) let us take $\delta =\delta ({\hat{u}}_{0},\epsilon )$, i.e., for $\hat{u}\in S$ such that $∥\hat{u}-{\hat{u}}_{0}{∥}_{c_{0}}\leq \delta$ we derive $∥\left(\tilde{F}\hat{u}\right)\left(t\right)-\left(\tilde{F}{\hat{u}}_{0}\right)\left(t\right){∥}_{c_{0}}\leq \epsilon$ for each t∈I. Moving forward,

$$\begin{array}{c} \begin{array}{cc} ∥\left(\Gamma \hat{u}\right)\left(t\right)-\left(\Gamma {\hat{u}}_{0}\right)\left(t\right){∥}_{c_{0}} & =\max\{|{\left(\Gamma \hat{u}\right)}_{ı}\left(t\right)-{\left(\Gamma {\hat{u}}_{0}\right)}_{ı}\left(t\right)|:\;ı\in J\}\\ & \leq \max\left\{\int_{0}^{t}\left|F_{ı}\left(s,u_{{ı}_{1}}\left(s\right),u_{{ı}_{2}}\left(s\right),\ldots \right)-F_{ı}\left(s,u_{{ı}_{1}}^{0}\left(s\right),u_{{ı}_{2}}^{0}\left(s\right),\ldots \right)\right|ds:\;ı\in J\right\}\\ & \leq T\epsilon , \end{array} \end{array}$$

which implies the desired claim.

Now, let us take the set $S_{1}=\Gamma S$. Recall that this set contains equicontinuous functions on *I*. In fact, taking an arbitrary $\hat{u}={\left(u_{ı}\right)}_{ı}\in S$, and bringing in mind our hypotheses, we conclude

$$\begin{array}{c} \begin{array}{cc} |{\left(\Gamma \hat{u}\right)}_{ı}\left(t\right)-{\left(\Gamma \hat{u}\right)}_{ı}\left(s\right)| & \leq |G_{ı}\left(t\right)-G_{ı}\left(s\right)|+|\int_{s}^{t}F_{ı}\left(\tau ,u_{{ı}_{1}}\left(\tau \right),u_{{ı}_{2}}\left(\tau \right),\ldots \right)d\tau |\\ & \leq |G_{ı}\left(t\right)-G_{ı}\left(s\right)|+|\int_{s}^{t}[\alpha_{ı}\left(\tau \right)+\beta_{ı}\left(\tau \right)\cdot \sup\{|u_{ȷ}\left(\tau \right)|:\;ı\leq ȷ\}]d\tau |\\ & \leq |G_{ı}\left(t\right)-G_{ı}\left(s\right)|+|\int_{s}^{t}\alpha_{ı}\left(\tau \right)d\tau |+|\int_{s}^{t}\beta_{ı}\left(\tau \right)\cdot \sup\{|u_{ȷ}\left(\tau \right)\left|:\;ı\leq ȷ\right\}d\tau |\\ & \leq |G_{ı}\left(t\right)-G_{ı}\left(s\right)|+|t-s|\sup\left\{\alpha_{ı}\left(t\right):\;t\in I\right\}+|\int_{s}^{t}\beta_{ı}\left(\tau \right)\cdot \left[a_{ı}\left(\tau \right)+b_{ı}\left(\tau \right)\right]d\tau |\\ & \leq |G_{ı}\left(t\right)-G_{ı}\left(s\right)|+|t-s|\left[\sup\left\{\alpha_{ı}\left(t\right):\;t\in I\right\}+\sup\left\{\beta_{ı}\left(t\right)\cdot \left[a_{ı}\left(t\right)+b_{ı}\left(t\right)\right]:\;t\in I\right\}\right]. \end{array} \end{array}$$

Since the function sequences $\left(\alpha_{ı}\left(t\right)\right),\left(\beta_{ı}\left(t\right)\right),\left(a_{ı}\left(t\right)\right)$, and $\left(b_{ı}\left(t\right)\right)$ are uniformly bounded on *I* and the function sequence $\left(G_{ı}\left(t\right)+\varphi_{ı}\right)$ is equicontinuous on *I*, from the above estimation, we conclude that the set $S_{1}=\Gamma S$ is equicontinuous on *I*.

Suppose that $S_{2}$ stands for $\mathrm{Conv}S_{1}$ (i.e., the closed convex hull of the set $S_{1}$). Clearly, $S_{2}$ is closed, bounded, and equicontinuous on *I*. Furthermore, $\Gamma S_{2}\subset S_{2}\subset S$.

From another point of view, for $\hat{u}\in S$, we get

$$\begin{array}{c} |{\left(\Gamma \hat{u}\right)}_{ı}\left(t\right)|\leq a_{ı}\left(t\right)+b_{ı}\left(t\right),\;ı\in J,\;t\in I. \end{array}$$

Since the sequence $\left(a_{ı}\left(t\right)+b_{ı}\left(t\right)\right)$ converges uniformly on *I* to the function vanishing identically on *I*, we conclude that for each ϵ>0, there exists an index ${ı}_{0}$ such that $|{\left(\Gamma \hat{u}\right)}_{ı}\left(t\right)|\leq \epsilon ,$ for each $ı\geq {ı}_{0}$ and for any t∈I. Therefore, by virtue of the criterion of compactness in the space $c_{0}$ as mentioned before, we infer that for each t∈I, the set $S_{1}\left(t\right)$ is relatively compact in the space $c_{0}$. The above arguments allow us to deduce that the set $S_{2}$ is relatively compact in the space $C(I,c_{0})$. Besides, the closedness of $S_{2}$ yields that it is compact. Hence, keeping in mind that Γ transforms continuously the set $S_{2}$ into itself, we result (by the Schauder fixed-point principle) that the operator Γ has a fixed point in the set $S_{2}$ being a solution of our problem. This completes the proof. □

**Remark** **1.** *In Theorem 3, if for some M>0*

(15) $$\begin{array}{c} |F_{ı}\left(t,u_{{ı}_{1}}\left(t\right),u_{{ı}_{2}}\left(t\right),\ldots \right)-F_{ı}\left(t,v_{{ı}_{1}}\left(t\right),v_{{ı}_{2}}\left(t\right),\ldots \right)|\leq M<T^{-1},\;ı\in J,\;t\in I, \end{array}$$

*then one can easily utilize the Banach contraction principle and find the unique solution for the subjected system.*

Theorem 3 implies the following result immediately.

**Theorem** **4.** *Suppose that all the conditions of Theorem 3 and *(15)* are satisfied. Then the problem *(1)* has a unique solution in $U_{I}$.*

## 7. Adomian Decomposition Method (ADM)

In this section we give standard description of the ADM to find the solution of Equation (1) in which we proved its existence in the last section. Consider the general equation

(16) $$\begin{array}{c} Lu+Ru+Nu=g \end{array}$$

where *u* is the function subjected to be found, L is the linear differential operator of higher order which is simply invertible. Suppose that its inverse is $L^{-1}$ and it will be an integral operator, *N* is the nonlinear operator, *R* is the remaining linear part and *g* is a given function (source). Taking $L^{-1}$ to both sides of (16) we get:

$$\begin{array}{c} L^{-1}\left(Lu+Ru+Nu=g\right)\;⟹\;L^{-1}Lu=L^{-1}g-L^{-1}N\left(u\right)-L^{-1}R\left(u\right), \end{array}$$

hence,

$$\begin{array}{c} u-\phi =L^{-1}g-L^{-1}N\left(u\right)-L^{-1}R\left(u\right), \end{array}$$

where ϕ is chosen from the initial conditions or from the boundary conditions or both, it depends on how we select differential operator that solve the given problem. The ADM considers that solution *u* of the functional equation can be decomposed into infinite series

$$\begin{array}{c} u=\sum_{n=0}^{\infty }u_{n} \end{array}$$

and the nonlinear term N(u) can be expressed as infinite series $Nu=\sum_{n=0}^{\infty }A_{n}$ where the $A_{n}$’s are the Adomian polynomials, which depend upon $u_{0},u_{1},\ldots ,u_{n}$. We recall that the Adomian polynomials $A_{n}$’s are first constructed by Adomian in 1992, he gave a general formula to determine the values of $A_{n}$’s:

$$A_{n}={\left[\frac{1}{n!}\frac{d^{n}}{d\lambda^{n}}N\left(\sum_{i=0}^{n}\lambda^{i}u_{i}\right)\right]}_{\lambda =0}.$$

Therefore, Equation (16) takes the following form:

(17) $$\begin{array}{c} \sum_{n=0}^{\infty }u_{n}=\phi +L^{-1}g-L^{-1}\sum_{n=0}^{\infty }A_{n}-L^{-1}\sum_{n=0}^{\infty }R\left(u_{n}\right). \end{array}$$

Now from Equation (17), we can derive the solution algorithm as follows:

(18) $$\begin{array}{c} \begin{array}{c} u_{0}=\phi +L^{-1}g,\;u_{n+1}=-L^{-1}\left(A_{n}+R\left(u_{n}\right)\right),\;n=0,1,2,\ldots . \end{array} \end{array}$$

Given $u_{0}$, the other terms of *u* can be defined, respectively. Hence, the existing solution *u* of Equation (16) can be determined by the series of recursive sequence $u_{n}$. To do this, let us rewrite the nonlinear differential Equation (13) as below

(19) $$\begin{array}{c} {\hat{u}}^{\prime }\left(t\right)=F\left(\hat{u}\right)+G\left(t\right). \end{array}$$

Assuming $\hat{u}=\sum_{n=0}^{\infty }{\hat{u}}_{n}$, and applying ADM we obtain

(20) $$\begin{array}{c} \begin{array}{c} {\hat{u}}_{0}=\phi +\int G\left(t\right)dt,\;{\hat{u}}_{n+1}=-L^{-1}\left(A_{n}\right),\;A_{n}={\left[\frac{1}{n!}\frac{d^{n}}{d\lambda^{n}}F\left(\sum_{i=0}^{n}\lambda^{i}{\hat{u}}_{i}\right)\right]}_{\lambda =0},\;n=0,1,2,\ldots . \end{array} \end{array}$$

where ϕ would be specified by the initial condition in (8). Moreover, the convergence of Adomian’s decomposition method is discussed in the Appendix A.

Since we have no information about the form of G(t,x) and the initial condition of the problem (1), we present a model to see how ADM works.

**Example** **1.** *In Equation *(1)*, let us suppose the non-homogeneity term $G\left(t,x\right)=t^{s}\ln{\left|x\right|}_{p},s\neq -1,$ and the initial condition $u\left(0,x\right)=\varphi \left(x\right):=\Omega (|x{|}_{p})$ which is the refinable function.*

*To formulate the function G in terms of basis $\psi_{k;jn}$, using the symbol $\xi :=p^{j}x-n$ we arrive at*

$$\begin{array}{c} \begin{array}{cc} G_{k;jn}\left(t\right)=\langle G\left(t,x\right),\psi_{k;jn}\left(x\right)\rangle & =\int_{Q_{p}}t^{s}\ln{\left|x\right|}_{p}\cdot \psi_{k;jn}\left(x\right)dx\\ & =t^{s}p^{\frac{-3j}{2}}\int_{Q_{p}}\ln(|p^{-j}{\left(\xi +n\right)|}_{p}{)\cdot \Omega (|\xi |}_{p})\cdot \chi_{p}\left(p^{-1}k\xi \right)d\xi \\ & =t^{s}p^{\frac{-3j}{2}}\int_{Q_{p}}\ln(p^{j}{\max\{|\xi |}_{p}{,|n|}_{p}\})\cdot {\Omega (|\xi |}_{p})\cdot \chi_{p}\left(p^{-1}k\xi \right)d\xi \\ & =t^{s}p^{\frac{-3j}{2}}{\left(j\ln p+\ln|n\right|}_{p})\int_{Q_{p}}{\Omega (|\xi |}_{p})\cdot \chi_{p}\left(p^{-1}k\xi \right)d\xi . \end{array} \end{array}$$

*For the case k=0 we get*

$$G_{0;jn}\left(t\right)=t^{s}p^{\frac{-3j}{2}}\ln p\cdot \left(j-{ord}_{p}\left(n\right)\right),$$

*and, otherwise, for the case 1≤k≤p−1, we have $G_{k;jn}\left(t\right)=0.$*

*On the other hand,*

$$\begin{array}{c} \begin{array}{cc} \varphi_{k;jn}=\langle \varphi \left(x\right),\psi_{k;jn}\left(x\right)\rangle & =\int_{Q_{p}}{\Omega (|x|}_{p})\cdot \psi_{k;jn}\left(x\right)dx\\ & =p^{\frac{-3j}{2}}\int_{Q_{p}}\Omega (|p^{-j}{\left(\xi +n\right)|}_{p}{)\cdot \Omega (|\xi |}_{p})\cdot \chi_{p}\left(p^{-1}k\xi \right)d\xi \\ & =p^{\frac{-3j}{2}}\int_{Q_{p}}\Omega (p^{j}{\max\{|\xi |}_{p}{,|n|}_{p}\})\cdot {\Omega (|\xi |}_{p})\cdot \chi_{p}\left(p^{-1}k\xi \right)d\xi \end{array} \end{array}$$

*where k=0,1,2,…,p−1 and $n\in Q_{p}/Z_{p}$. Considering ${\left|n\right|}_{p}=p^{-\gamma }$ for some integer γ≤−1 together with the fact that $\Omega (|\xi {|}_{p})\neq 0$ if and only $\xi \in S_{r}$ for some r≤0, we obtain*

$$\begin{array}{c} \begin{array}{cc} \varphi_{k;jn} & =p^{\frac{-3j}{2}}\Omega \left(p^{j-\gamma }\right)\sum_{r\leq 0}\int_{S_{r}}\chi_{p}\left(p^{-1}k\xi \right)d\xi \\ & =p^{\frac{-3j}{2}}\Omega \left(p^{j-\gamma }\right)\sum_{r\leq 0}\left(\int_{B_{r}}\chi_{p}\left(p^{-1}k\xi \right)d\xi -\int_{B_{r-1}}\chi_{p}\left(p^{-1}k\xi \right)d\xi \right). \end{array} \end{array}$$

*If k=0 then*

$$\varphi_{0;jn}=p^{\frac{-3j}{2}}\Omega \left(p^{j-\gamma }\right)=p^{\frac{-3j}{2}}\Omega \left(p^{j-{ord}_{p}\left(n\right)}\right).$$

*Otherwise, for the case 1≤k≤p−1, using the fact that*

$$\begin{array}{c} \int_{B_{r}}\chi_{p}\left(\xi x\right)dx=\left\{\begin{array}{c} p^{r},\;{\left|\xi \right|}_{p}\leq p^{-r},\\ {0,\;|\xi |}_{p}\geq p^{-r+1},\;r\in Z, \end{array}\right. \end{array}$$

*we see that*

$$\begin{array}{c} \begin{array}{c} \varphi_{k;jn}=p^{\frac{-3j}{2}}\Omega \left(p^{j-{ord}_{p}\left(n\right)}\right)\left(-p^{-1}+\sum_{r\leq -1}\left(p^{r}-p^{r-1}\right)\right)=0. \end{array} \end{array}$$

*On the other hand, looking at *(11)* one can see that*

$$\begin{array}{c} F_{ı}\left(\hat{u}\right)=\sum_{{ı}^{\prime }\in J}f_{ı}\left({ı}^{\prime }\right)\cdot {\left|u_{{ı}^{\prime }}\right|}^{2},\;{ı}^{\prime }:=\left(k^{\prime },j^{\prime },n^{\prime }\right),\;ı:=\left(k,j,n\right) \end{array}$$

*where*

$$\begin{array}{c} f_{ı}\left({ı}^{\prime }\right)=\int_{Q_{p}}p^{1-j^{\prime }}\Omega (|p^{j^{\prime }}x-n^{\prime }{|}_{p})\psi_{ı}\left(x\right)dx. \end{array}$$

*Hence,*

$$\begin{array}{c} F\left(\hat{u}\right)={\left(\sum_{{ı}^{\prime }\in J}f_{ı}\left({ı}^{\prime }\right)\cdot {\left|u_{{ı}^{\prime }}\right|}^{2}\right)}_{ı}-\theta \cdot p^{2(1-j)}\hat{u}. \end{array}$$

*Now, we are ready to apply ADM as follows.*

$${\hat{u}}_{0}={\left(u_{0,ı}\right)}_{ı}=\phi +\int G\left(t\right)dt=\left\{\begin{array}{c} \phi +\frac{t^{s+1}}{s+1}p^{\frac{-3j}{2}}\ln p\cdot \left(j-{ord}_{p}\left(n\right)\right),\;k=0,\\ 0,\;1\leq k\leq p-1. \end{array}\right.$$

(21) $$\begin{array}{c} \begin{array}{c} {\hat{u}}_{n+1}=-L^{-1}\left(A_{n}\right),\;A_{n}={\left[\frac{1}{n!}\frac{d^{n}}{d\lambda^{n}}F\left(\sum_{i=0}^{n}\lambda^{i}{\hat{u}}_{i}\right)\right]}_{\lambda =0},\;n=0,1,2,\ldots . \end{array} \end{array}$$

*The first three terms of $A_{n}$’s are*

$$\begin{array}{cc} A_{0}={\left[\frac{1}{0!}\frac{d^{0}}{d\lambda^{0}}F\left(\lambda^{0}{\hat{u}}_{0}\right)\right]}_{\lambda =0}\; & =F({\hat{u}}_{0})\\ & ={\left(\sum_{\left(0,j^{\prime },n^{\prime }\right)}f_{ı}\left(0,j^{\prime },n^{\prime }\right)\cdot |\phi +\frac{t^{s+1}}{s+1}p^{\frac{-3j^{\prime }}{2}}\ln p\cdot \left(j^{\prime }-{ord}_{p}\left(n^{\prime }\right)\right){|}^{2}\right)}_{ı}-\theta \cdot p^{2(1-j)}{\hat{u}}_{0}, \end{array}$$

$$\begin{array}{c} \begin{array}{cc} A_{1} & ={\left[\frac{1}{1!}\frac{d}{d\lambda }F\left(\sum_{i=0}^{1}\lambda^{i}{\hat{u}}_{i}\right)\right]}_{\lambda =0}\\ & ={\hat{u}}_{1}F^{\prime }\left({\hat{u}}_{0}\right)=-2\left(\int A_{0}\left(t\right)dt\right)(\sum_{\left(0,j^{\prime },n^{\prime }\right)}f_{ı}\left(0,j^{\prime },n^{\prime }\right)\cdot \left(\phi +\frac{t^{s+1}}{s+1}p^{\frac{-3j^{\prime }}{2}}\ln p\cdot \left(j^{\prime }-{ord}_{p}\left(n^{\prime }\right)\right)\right)\\ & \times \left(t^{s}p^{\frac{-3j^{\prime }}{2}}\ln p\cdot \left(j^{\prime }-{ord}_{p}\left(n^{\prime }\right)\right)\right){)}_{ı}-{\left(\theta \cdot p^{2(1-j)}\right)}_{ı}, \end{array} \end{array}$$

$$\begin{array}{c} \begin{array}{cc} A_{2} & ={\left[\frac{1}{2!}\frac{d^{2}}{d\lambda^{2}}F\left(\sum_{i=0}^{2}\lambda^{i}{\hat{u}}_{i}\right)\right]}_{\lambda =0}=\frac{{\hat{u}}_{1}^{2}}{2!}F^{\prime \prime }\left({\hat{u}}_{0}\right)+{\hat{u}}_{2}F^{\prime }\left({\hat{u}}_{0}\right),\;\cdots \end{array} \end{array}$$

*We recall that the ADM is analogous to find the Taylor’s series expansion for the nonlinear function $F(\hat{u})$ around the initial function ${\hat{u}}_{0}$. Following this way and finding the Adomian polynomials $A_{n}$, from *(21)* we get the solution as form of $\hat{u}=\sum_{n=0}^{\infty }{\hat{u}}_{n}.$ Moreover, using the value of $\varphi_{k;jn}$ as initial value, the constant ϕ would be determined.*

## 8. Concluding Remarks

The use of tree-like (ultrametric) geometry is the promising direction in modeling of fluids’ transport through capillary networks in porous disordered media. Such geometry approximates fractal (and multi-fractal) structures in Geo-conduits.

Theory of linear dynamical equations (especially, *p*-adic) is well developed and its application to geophysics (see [36]) did not demand essential mathematical efforts. However, as well as in Euclidean geophysical modeling, the basic ultrametric dynamical equations are nonlinear. One of such equations, an analog of the Navier–Stokes equation, was derived in recent paper [38]. Its study posed a variety of new problems. This study is especially complicated in the absence of the general theory of nonlinear (pseudo-)differential equations on *p*-adic spaces; just the first steps in this direction were done in articles [37,43]. The present paper is the important step towards establishing theory *p*-adic Navier–Stokes equation.

Fractal and multifractal mathematical models are widely used for diagnostic of hydrocarbon-reservoirs stratigraphic patterns anisotropy (see, e.g., [44,45]). The concrete “on field applications” are based on software; one of the promising complexes of diagnostic programs was developed by the research group of K. Oleschko. This complex was actively used in realization of the projects for Mexican oil-industry, e.g., the project SENER-CONACYT-Hidrocarburos, Yacimiento petrolero como un reactor fractal. Development of ultrametric models for oil transport plays the important role in mathematical justification of application of (multi-)fractal models for Mexican Petroleum Industry.

## Appendix A. (Convergence of Adomian’s Decomposition Method)

The convergence of ADM has been investigated by various authors. One significant fact is that the series solution of Adomian’s decomposition technique often converges very fast to the exact solution, if there is only one, and to one of the solutions, if several exist, tending the general term of the series solution to zero very fast, as 1/(mq)!, for *m* terms and the *q*th order of the linear operator *L* (see [66,70]).

Suppose the Hilbert space $H=L^{2}\left(I_{1}\times I_{2}\right)$ defined by the set of applications

$$u:I_{1}\times I_{2}\to R,\;\mathrm{with}\;\int_{I_{1}\times I_{2}}u^{2}\left(s,t\right)dsdt<\infty .$$

In Equation (16) let us consider the operator Tu=Ru+Nu, be hemicontinuous and satisfy the following hypothesis:

(a) $\langle T\left(u\right)-T\left(v\right),u-v\rangle \geq {k∥u-v∥}^{2},\;k>0,\forall u,v\in H;$
(b) ∀M>0,∃C(M)>0,∥u∥≤M,∥v∥≤M⇒〈T(u)−T(v),w〉≤C(M)∥u−v∥∥w∥,∀w∈H.

If the above assumptions are fulfilled, the Adomian’s method is convergent (see [71,72,73]). Replacing Tu:=F(u)+G, the following are sufficient conditions of convergence of ADM to the Equation (19),

(c) $\langle F\left(u\right)-F\left(v\right),u-v\rangle \geq {k∥u-v∥}^{2},\;k>0,\forall u,v\in H;$
(d) ∀M>0,∃C(M)>0,∥u∥≤M,∥v∥≤M⇒〈F(u)−F(v),w〉≤C(M)∥u−v∥∥w∥,∀w∈H.
